# Infection Dynamics of Cotransmitted Reproductive Symbionts Are Mediated by Sex, Tissue, and Development

**DOI:** 10.1128/aem.00529-22

**Published:** 2022-06-22

**Authors:** Megan W. Jones, Laura C. Fricke, Cody J. Thorpe, Lauren O. Vander Esch, Amelia R. I. Lindsey

**Affiliations:** a Department of Entomology, University of Minnesota, St. Paul, Minnesota, USA; University of Queensland

**Keywords:** *Wolbachia*, cytoplasmic incompatibility, symbiosis, vertical transmission, coinfection

## Abstract

One of the most prevalent intracellular infections on earth is with *Wolbachia*, a bacterium in the *Rickettsiales* that infects a range of insects, crustaceans, chelicerates, and nematodes. *Wolbachia* is maternally transmitted to offspring and has profound effects on the reproduction and physiology of its hosts, which can result in reproductive isolation, altered vectorial capacity, mitochondrial sweeps, and even host speciation. Some populations stably harbor multiple *Wolbachia* strains, which can further contribute to reproductive isolation and altered host physiology. However, almost nothing is known about the requirements for multiple intracellular microbes to be stably maintained across generations while they likely compete for space and resources. Here, we use a coinfection of two *Wolbachia* strains (“*w*Ha” and “*w*No”) in Drosophila simulans to define the infection and transmission dynamics of an evolutionarily stable double infection. We find that a combination of sex, tissue, and host development contributes to the infection dynamics of the two microbes and that these infections exhibit a degree of niche partitioning across host tissues. *w*Ha is present at a significantly higher titer than *w*No in most tissues and developmental stages, but *w*No is uniquely dominant in ovaries. Unexpectedly, the ratio of *w*Ha to *w*No in embryos does not reflect those observed in the ovaries, indicative of strain-specific transmission dynamics. Understanding how *Wolbachia* strains interact to establish and maintain stable infections has important implications for the development and effective implementation of *Wolbachia*-based vector biocontrol strategies, as well as more broadly defining how cooperation and conflict shape intracellular communities.

**IMPORTANCE** *Wolbachia* is a maternally transmitted intracellular bacterium that manipulates the reproduction and physiology of arthropods, resulting in drastic effects on the fitness, evolution, and even speciation of its hosts. Some hosts naturally harbor multiple strains of *Wolbachia* that are stably transmitted across generations, but almost nothing is known about the factors that limit or promote these coinfections, which can have profound effects on the host’s biology and evolution and are under consideration as an insect-management tool. Here, we define the infection dynamics of a known stably transmitted double infection in Drosophila simulans with an eye toward understanding the patterns of infection that might facilitate compatibility between the two microbes. We find that a combination of sex, tissue, and development all contributes to infection dynamics of the coinfection.

## INTRODUCTION

Eukaryotic cells are home to a diversity of intracellular microbes including mitochondria, plastids, symbionts, and pathogens, many of which are vertically inherited via the maternal germ line. The community and interactions between intracellular microbes are associated with diverse effects on host physiology and health. Despite the importance of the intracellular community, little is known about the factors that promote, inhibit, or regulate the establishment and transmission of multiple, coinfecting, intracellular microbes.

Arthropods are particularly rich in examples of such infections. It is estimated that more than half of arthropods have at least one heritable bacterial symbiont, and ~12% have two or more of these infections (1, 2). The most common of these is an alphaproteobacterium, *Wolbachia*, a close relative of the intracellular human pathogens *Anaplasma*, *Rickettsia*, and *Ehrlichia* (3). Unlike their close relatives, *Wolbachia* inhabits the cells of arthropods and nematodes, is primarily vertically transmitted via the maternal germ line, and alters host physiology and reproduction to facilitate spread through a population (4, 5). Some arthropods stably harbor multiple coinfecting *Wolbachia* strains (6–13), resulting in drastic effects on host fitness, gene flow between populations, horizontal transfer between *Wolbachia* strains, and even host speciation (8, 10, 14–18). Not only are *Wolbachia* coinfections significant for evolution of both the microbes and the arthropod host, but the increasing interest in establishing secondary *Wolbachia* infections for use in insect control programs necessitates a mechanistic investigation of these intracellular inhabitants (19–21). Previous successes in *Wolbachia*-mediated vector control were more easily attainable because key vector species such as Aedes aegypti so happened to naturally lack *Wolbachia* (22, 23). However, many other pest and vector species are already infected with resident *Wolbachia* strains, and establishment of a secondary infection is a potential avenue for control methods (20, 21, 24). Furthermore, pathogens and symbionts in related systems are rarely in complete isolation and the intracellular interactions between symbiotic microbes, pathogenic microbes, mitochondria, and viruses can all contribute to altered host physiology, vector competence, and/or clinical progression of disease (25–30).

While very little is known about the infection dynamics of cooccurring *Wolbachia* strains, there are several shared characteristics across many of the naturally occurring *Wolbachia* coinfections, indicating there may be shared mechanisms and selective pressures at play. For example, in Aedes albopictus infected with *w*AlbA and *w*AlbB *Wolbachia* strains (10), Nasonia vitripennis (with *w*VitA and *w*VitB [7]), Dactylopius coccus (with *w*DacA and *w*DacB [31]), and Drosophila simulans (with *w*Ha and *w*No [15]), each insect has one *Wolbachia* strain from supergroup A and one from supergroup B, perhaps indicating that more divergent strains are more compatible in a coinfection, maybe as a result of niche partitioning. In support of this idea, a recent study describing an artificially generated triple infection of *Wolbachia* strains in Aedes albopictus showed there was strong competition between *Wolbachia* strains from the same supergroup but not between *Wolbachia* strains from different supergroups (32). However, there are insects that harbor double infections of strains originating from the same supergroup, such as the butterfly Eurema hecabe, host to a variety of B-supergroup strains (33). And, some hosts have three stably infecting *Wolbachia* strains, two from supergroup B and one from supergroup A (e.g., the adzuki bean beetle with *w*BruCon, *w*BruOri, and *w*BruAus [12] and a lepidopteran, Homona magnanima, with *w*Hm-a, -b, and -c [34]). Niche partitioning might not require phylogenetic distance but could result from other physiological differences between strains. Indeed, there are other examples of artificially generated multiple infections, but the outcomes are highly variable: sometimes the infection destabilizes and is quickly lost, and at other times it is stable across many generations (35–40). Ultimately, we do not know which factors facilitate successful establishment and transmission of multiple *Wolbachia* strains within one host matriline.

There is literature that suggests the titers of individual strains are differentially regulated. In Aedes albopictus mosquitoes, the native *w*AlbB strain is present at ~6× the titer of the coinfecting native *w*AlbA strain (9). In Drosophila simulans, the *w*Ha and *w*No strains establish themselves at different titers under monoinfection conditions, and these titers depend on the combination of strain identity and host tissue (41, 42). However, studies that investigated these strain-specific dynamics leveraged independent fly genetic backgrounds that carried either the *w*Ha strain or the *w*No strain, which confounds our interpretation of coinfection dynamics (15, 41–43).

Broadly, there is evidence for both (i) host control over the titer of individual *Wolbachia* strains and/or (ii) the presence of a coinfecting strain contributing to the regulation of *Wolbachia* density (11, 44). However, we have limited knowledge of (i) how coinfecting strains might establish themselves across host tissues and developmental stages, (ii) whether coinfecting strains facilitate each other’s transmission, (iii) whether strains evolved to occupy unique niches within the host, (iv) whether strains go through different severities of population bottleneck from ovary to oocyte, (v) whether there are combinatorial effects of the coinfection on host physiology, and ultimately, (vi) the host and microbial mechanisms that regulate the maintenance of these coinfections. To begin to investigate these questions, we explore infection and transmission dynamics of multiple vertically inherited intracellular symbionts in a Drosophila simulans model which naturally harbors a stable coinfection of two *Wolbachia* strains: *w*Ha and *w*No.

## RESULTS

### Coinfecting strains *w*Ha and *w*No share 75% of their coding sequences.

**FIG 1:**
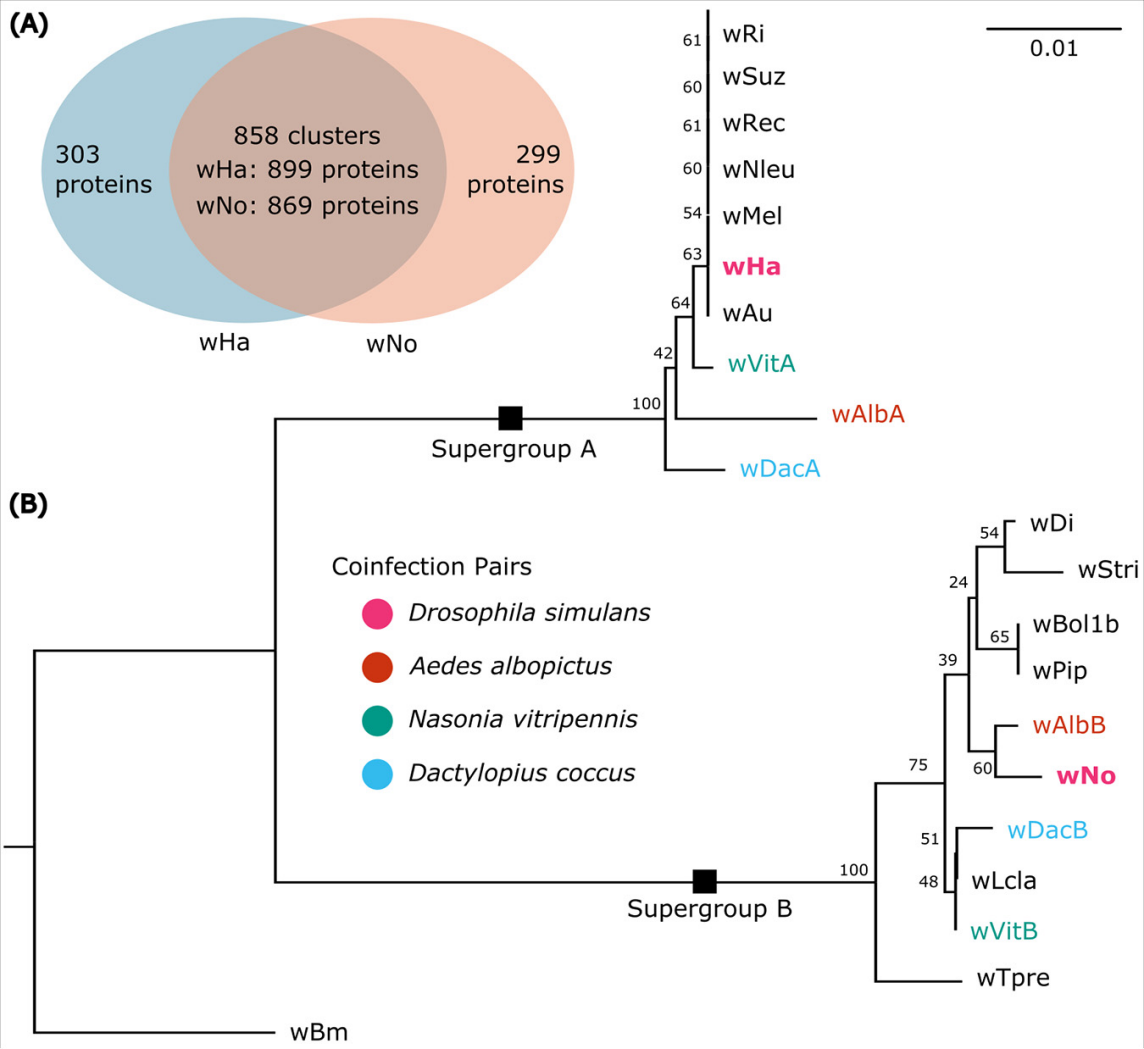
Evolution of coinfecting *w*Ha and *w*No. (A) Shared and unique genes between the focal strains *w*Ha and *w*No that coinfect Drosophila simulans. (B) Phylogenetic reconstruction of A- and B-supergroup *Wolbachia* strains based on FtsZ protein sequences, with colors indicating pairs of *Wolbachia* strains that can be found together within a given host. Node labels indicate bootstrap support (*n* = 100 replicates). Focal strains *w*Ha and *w*No are bolded.

To better understand the factors that might facilitate compatibility of two strains, we used a suite of bioinformatic approaches to look at phylogenetic and genomic patterns of *Wolbachia* coinfections. Our focal strains, *w*Ha and *w*No (from supergroups A and B, respectively), which coinfect some populations of Drosophila simulans, share 858 orthologous groups of proteins, approximately 75% of the coding content of each strain (Fig. 1A). The remaining proteins in each strain that are not shared are largely hypothetical, unannotated protein sequences, and only 10 to 15% were assigned a putative function (*w*Ha, *n* = 31/303; *w*No, *n *= 44/299). Annotated proteins (i.e., those assigned a KEGG KO term) specific to *w*No included 16 transposases and 15 proteins that were related to transcription, DNA repair, or endonuclease activity, and the remaining proteins were largely metabolic in predicted function (see Table S2 in the supplemental material). Notably, *w*No encodes a putative multidrug efflux pump that is not present in *w*Ha. *w*Ha-specific proteins included 15 transposases, three proteins predicted to be involved in transcription or DNA repair, and then a suite of proteins mostly with predicted functions in amino acid transport and metabolism. Interestingly, the *w*Ha strain has two proteins for an addiction module toxin (RelE/StbE family) and a predicted eukaryote-like golgin-family protein, potentially an effector protein that could interact with host intracellular membranes.

### Strain-specific titers are sex dependent.

**FIG 2:**
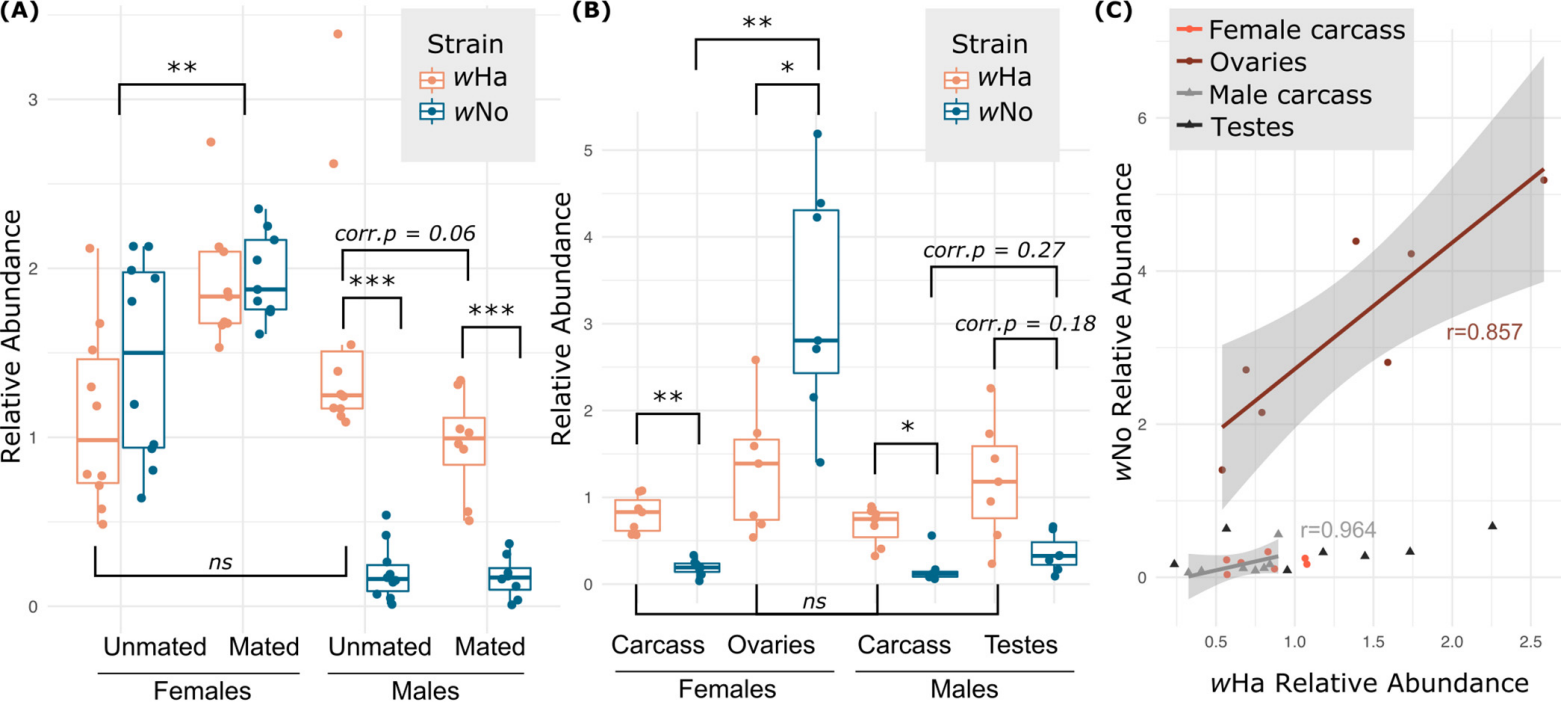
Infection densities of coinfecting *Wolbachia* strains. (A) *w*Ha and *w*No titers in whole-body females (unmated, *n* = 10; mated, *n* = 9) and males (unmated, *n* = 10; mated, *n* = 8). There was a significant effect of the interaction between fly sex and mated status (F_1,36_ = 4.076, *P* = 0.033) and sex (F_1,36_ = 69.568, *P* = 0.001) but not mated status alone (F_1,36_ = 0.488, *P* = 0.526) on the coinfection. (B) *w*Ha and *w*No titers of gonads and carcasses of unmated males and females (*n* = 7 paired gonad-carcass samples for each sex). The interaction of sex and tissue significantly affected the coinfection (F_1,27_ = 19.334, *P* = 0.001), as well as sex alone and tissue alone (F_1,27_ = 19.982, *P* = 0.001, and F_1,27_ = 27.147, *P* = 0.001, respectively). (C) Correlation between *w*Ha and *w*No relative abundances within each sample. Regression lines are shown for ovaries and male carcasses, for which we identified significant correlations in strain-specific relative abundance (see text). Across all panels, *post hoc* testing with Mann-Whitney U (two groups) or Kruskal-Wallis rank sum (>2 groups) test gave the following significance: ***, corrected *P* < 0.001; **, corrected *P* < 0.01; *, corrected *P* < 0.05; ns, not significant. Corrected *P* values are indicated for comparisons where *P* was <0.05 prior to Bonferroni corrections but was >0.05 after correction for multiple comparisons. See Table S8 in the supplemental material for all *post hoc* statistics.

We assessed the titers of the *w*Ha and *w*No strains in whole-body 3-day-old unmated males and females and 3-day-old males and females 2 days postmating (Fig. 2A). There was a significant effect of the interaction between fly sex and mated status (F_1,33_ = 4.076, *P* = 0.033) as well as a significant effect of sex alone (F_1,33_ = 69.568, *P* = 0.001) but not of mated status alone (F_1,33_ = 0.488, *P* = 0.500). This was seen as relatively equal titers of *w*Ha and *w*No in female flies that increased in relative abundance upon mating (corrected *P* [corr.*P*] = 0.0079). In contrast, males had drastically reduced titers of *w*No, both relative to *w*No in females (corr.*P* = 0.0025) and relative to the coinfecting *w*Ha strain within a male (unmated, corr.*P* = 0.0001; mated, corr.*P* = 0.0009). Relative *w*Ha titers in unmated males were not significantly different from relative *w*Ha titers in unmated females (*P* = 0.1823), but there was a slight reduction in relative *w*Ha titer in males upon mating (corr.*P* = 0.0666). Together, these data indicate strong sex- and mating-dependent effects on coinfection dynamics.

### Coinfection dynamics are sex and tissue dependent.

A subset of the unmated males and females were dissected prior to DNA extraction, resulting in paired gonadal and “carcass” (all remaining tissue) samples for each fly. Strain-specific quantitative PCR (qPCR) revealed that the interaction of sex and tissue identity had a significant effect on the relative abundance of the two strains in the coinfection (F_1,27_ = 19.334, *P* = 0.001). Additionally, there was a significant effect of sex alone and tissue alone (F_1,27_ = 19.982, *P* = 0.001, and F_1,27_ = 27.147, *P* = 0.001, respectively). In contrast to the relatively equal titers of *w*Ha and *w*No seen in whole female samples (Fig. 2A), we found that ovaries were highly enriched for the *w*No strain (corr.*P* = 0.0489) (Fig. 2B). This is in strong contrast to the patterns of *w*Ha, which was not significantly different between tissue types (*P* = 0.2248) but was higher than *w*No relative abundance in all nonovary samples, often quite significantly so (female carcass, corr.*P* = 0.0041; male carcass, corr.*P* = 0.0163; testes, corr.*P* = 0.1835).

We then tested for correlation between the relative abundances of *w*Ha and *w*No within a sample type. We found that in ovaries and male carcasses, there was a significant positive correlation between the relative abundances of *w*Ha and *w*No (rho = 0.0238, *P* = 0.8571, and rho = 0.9643, *P* = 0.0023, respectively). However, in testes and female carcasses, titers of *w*Ha and *w*No were uncorrelated (rho = 0.0714, *P* = 0.9063, and rho = 0.5357, *P* = 0.2357, respectively). Next, we asked if there was any correlation in the coinfection between samples that originated from the same fly. We did this in three ways: (i) by comparing the relative abundances of a strain between carcass and gonads, (ii) by comparing the ratio of *w*Ha and *w*No within the gonads to the same ratio in the carcass, and (iii) by comparing the relative abundances of *w*Ha and *w*No between gonads and carcass. In all cases, we found no significant relationship between the infection dynamics in the gonads and the carcass (Fig. S1). In fact, female flies had a very consistent ratio of *w*Ha to *w*No in the ovaries (0.39 ± 0.1) and highly variable *w*Ha/*w*No ratios in the carcass (6.08 ± 4.69). In agreement with the data shown in Fig. 2B, the opposite is true in males: the *w*Ha/*w*No ratio is more consistent in the carcass but highly variable in the testes (Fig. S1).

### The coinfection is dynamic across development.

**FIG 3:**
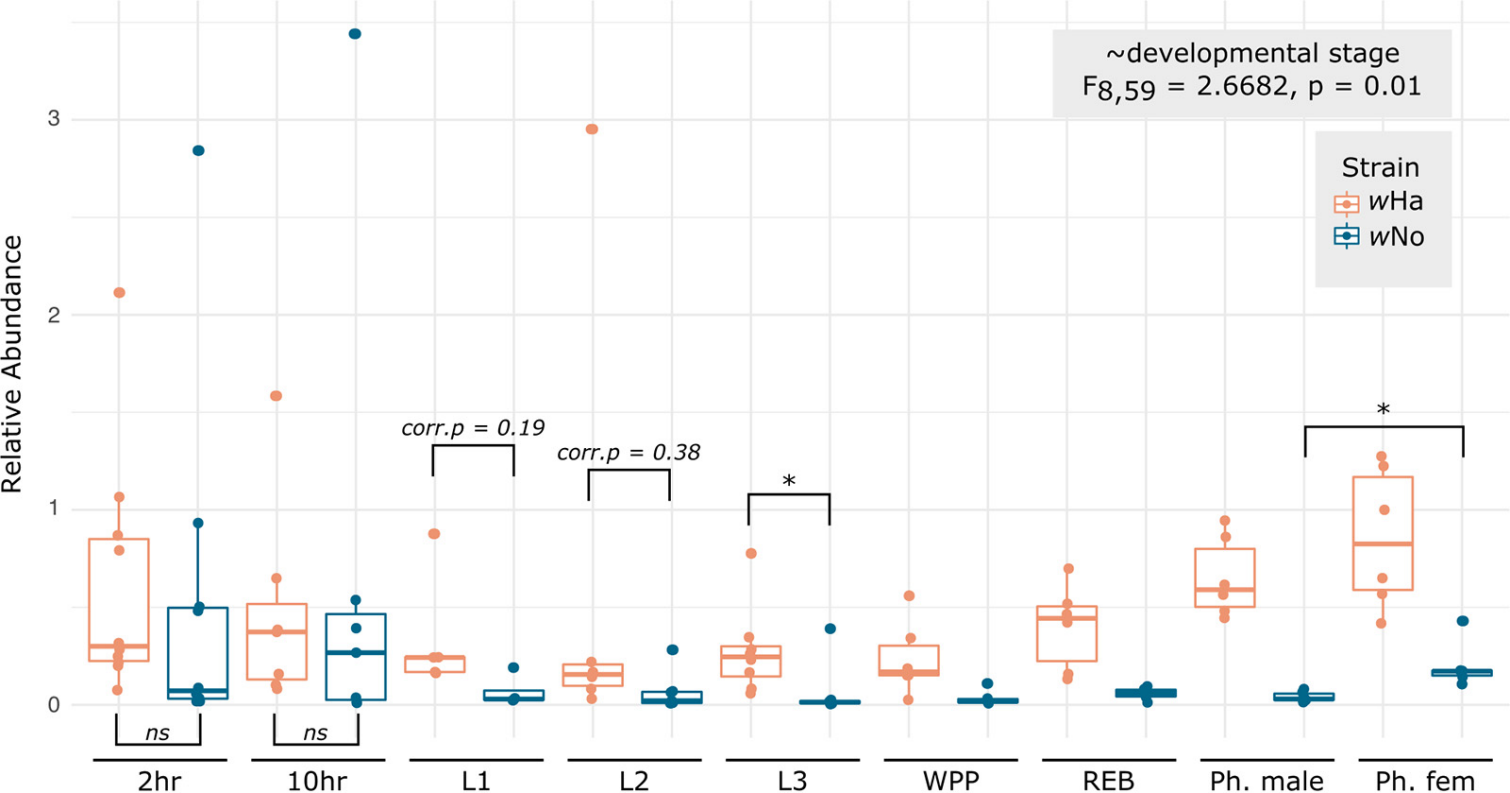
Coinfection is dynamic across development. Relative abundance of *w*Ha and *w*No across development. Developmental stages include, from left to right, 2-h-old embryos (*n* = 10), 10-h-old embryos (*n* = 7), first-instar larvae (L1, *n* = 5), second-instar larvae (L2, *n* = 6), third-instar larvae (L3, *n* = 8), white prepupae (WPP, *n* = 6), red-eye bald pupal stage (REB, *n* = 6), pharate (Ph.) males (*n* = 6), and Ph. females (*n* = 6). *Post hoc* testing with Mann-Whitney U gave the following significance: *, corrected *P* < 0.05; ns, not significant. Corrected *P* values are indicated for comparisons where *P* was <0.05 prior to Bonferroni corrections but >0.05 after correction for multiple comparisons. See Table S8 in the supplemental material for all *post hoc* statistics.

Given the difference in coinfection between sexes and tissues, we wondered if this was due to differences in transmission of *Wolbachia* to embryos and/or changes across development. To test this, we set up timed egg-lays and collected a developmental series that included seven time points across development (from 2-h-old embryos to red-eye bald pupal stage) as well as newly emerged pharate males and females (Fig. 3). Strain-specific qPCR revealed that the coinfection changed significantly across development (Fig. 3; F_8,59_ = 2.6682, *P* = 0.01). Notably, the pattern of infection in very young embryos did not resemble any of the previously assessed sample types, including the ovaries. Indeed, in 2-h-old embryos there was no significant difference in the relative abundances of *w*Ha and *w*No (*P* = 0.1655), unlike the strong *w*No bias in ovaries, and unlike the strong *w*Ha bias in carcasses and testes. Across larval development, the coinfection converged on a pattern more similar to the carcass tissue and testes, where *w*Ha titers were significantly higher than *w*No (L3, corr.*P* = 0.0419). In the newly eclosed pharate females, there was a significant increase in *w*No titer relative to the pharate males (corr.*P* = 0.0130), likely indicative of a shift toward the *w*No bias we saw in 3-day-old female ovaries (Fig. 2B).

### Transmission of the coinfection to embryos is strain specific.

**FIG 4:**
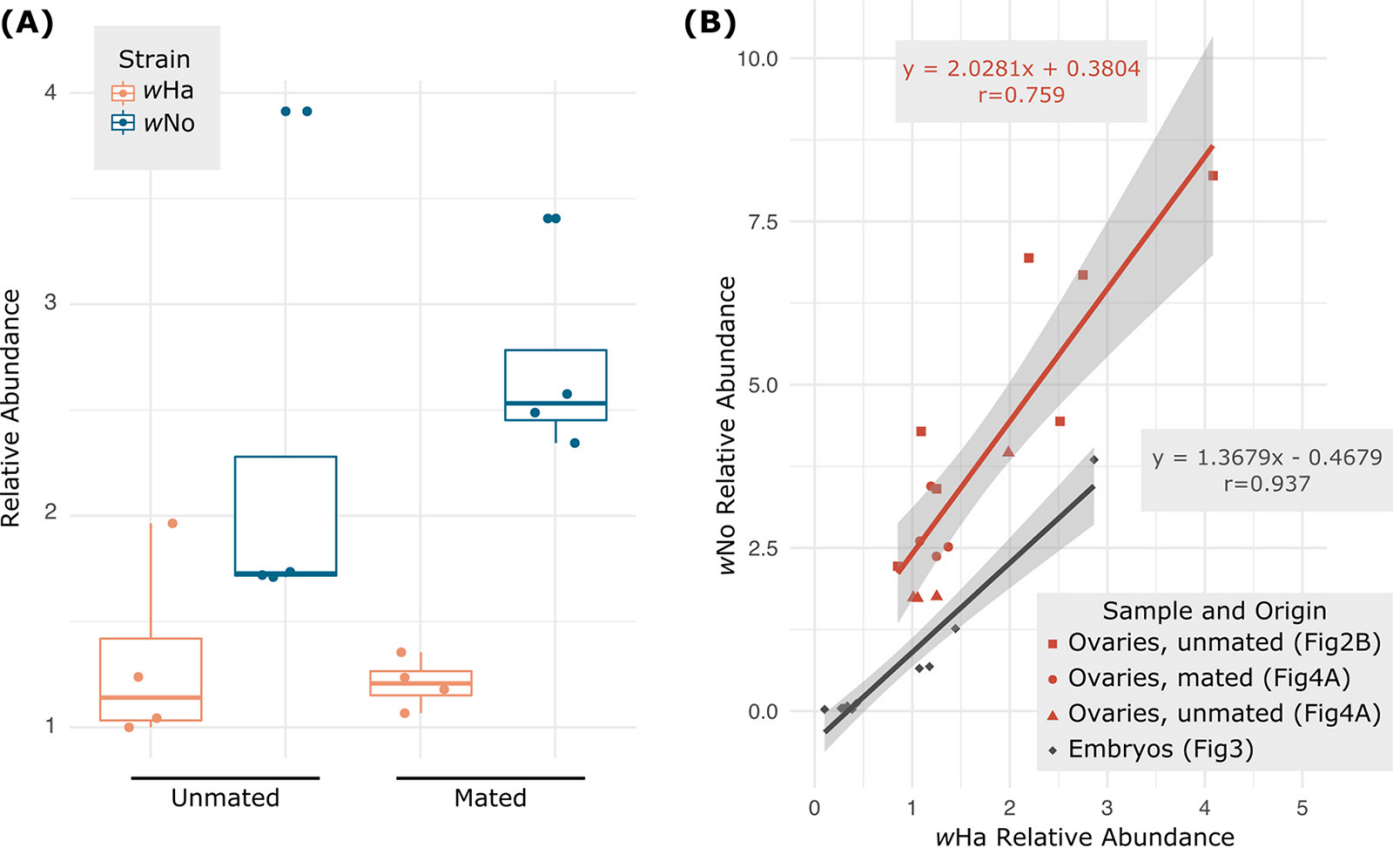
The ratio of *w*Ha and *w*No transmitted to embryos is not reflective of the coinfection in ovaries. (A) Titers of *w*Ha and *w*No do not significantly change upon mating. Newly eclosed females were collected, and a subset were mated after 24 h. Three days posteclosion, ovaries were dissected from the mated and unmated females (*n* = 8 each). Only strain identity (*w*Ha versus *w*No) significantly affected titer (~strain*mated status, F_1,12_ = 1.055, *P* = 0.3246; ~mated status, F_1,12_ = 0.473, *P* = 0.5049; ~strain, F_1,12_ = 22.891, *P* = 0.0005). (B) *w*Ha and *w*No titers are strongly correlated within ovaries and within embryos. However, the ratios of *w*Ha to *w*No are significantly different between the two, indicated by the negative *y*-intercept (*w*No) for embryos compared to ovaries.

The developmental series revealed that very young embryos had coinfections that were dissimilar to the infections in ovaries, which raises questions about how the two *Wolbachia* strains are transmitted to the next generation (Fig. 2B). However, the data presented in Fig. 2B were generated from unmated females, so we sought to determine if the coinfection differed due to mating, which might explain why the embryos had differing ratios of the two *Wolbachia* strains. We found no significant difference in the coinfection between ovaries derived from 3-day-old mated and unmated females, and in both cases *w*No had a significantly higher titer than *w*Ha (Fig. 4A; ~strain*mated status, F_1,12_ = 1.055, *P* = 0.3246; ~mated status, F_1,12_ = 0.473, *P* = 0.5049; ~strain, F_1,12_ = 22.891, *P* = 0.0005). We then used linear regression to assess the relationship between *w*Ha and *w*No in ovary and embryo samples with an eye toward the transmission dynamics. In both sample types there was a significant positive correlation between *w*Ha and *w*No (ovaries, F_1,13_ = 45.13, *P* < 0.0001, *r* = 0.759; embryos, F_1,8_ = 133.9, *P* < 0.0001, *r* = 0.937). However, in ovaries *w*No was more than double the relative abundance of *w*Ha, whereas the two infections were closer to 1:1 in embryos (Fig. 4B; ovaries, *y* = 2.0281*x* + 0.3804; embryos, *y* = 1.3679*x* − 0.4679). Therefore, transmission to embryos favors *w*Ha. This is also seen in the negative intercept along the *y* axis (*w*No), indicating a higher likelihood that embryos might receive only *w*Ha but not *w*No at especially low levels of overall transmission, even though ovaries contain double the titer of *w*No.

### Heat stress facilitates destabilization of cotransmission.

**FIG 5:**
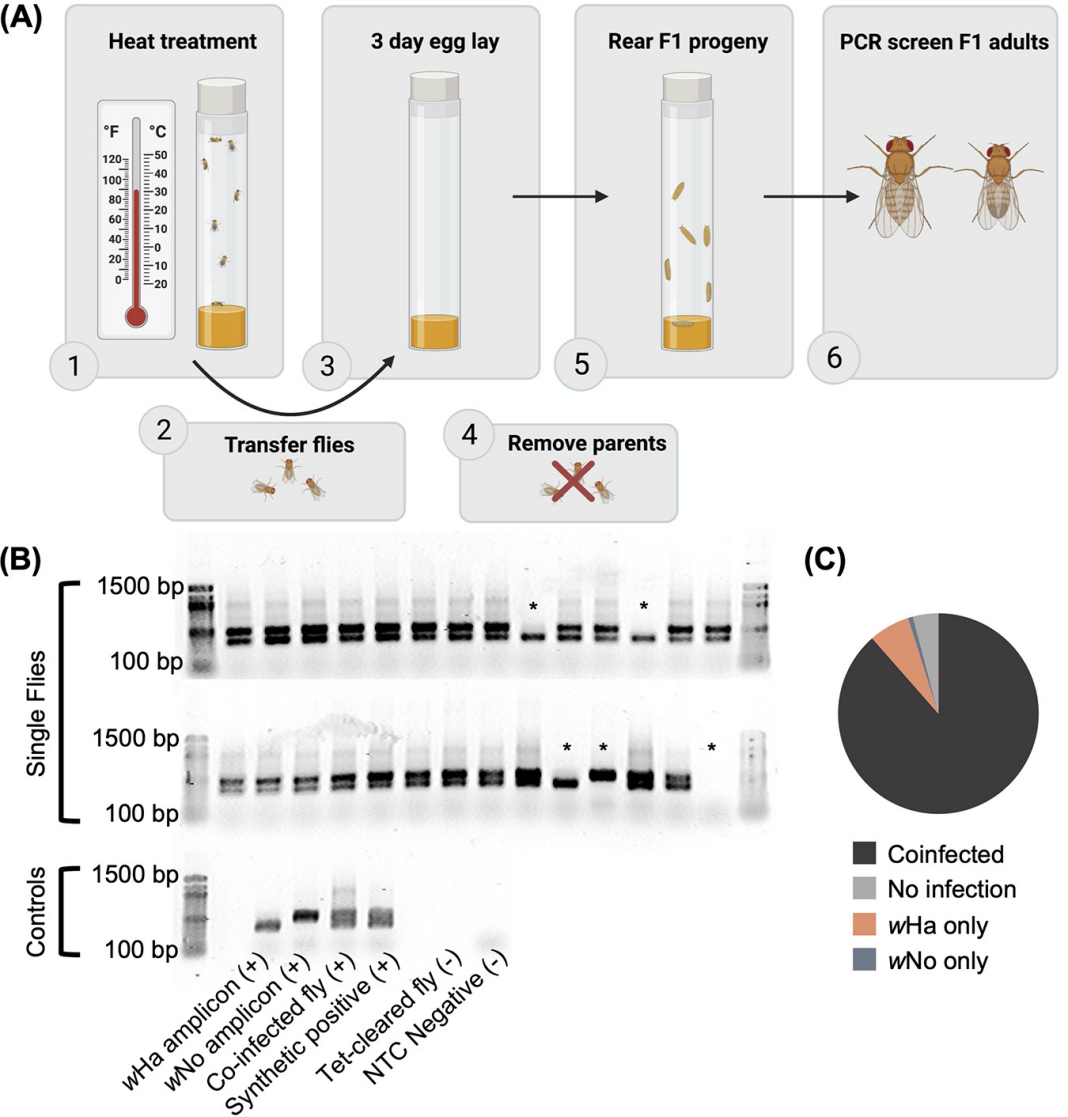
Heat stress destabilizes cotransmission of *w*Ha and *w*No. (A) Schematic of heat-curing experimental design created with BioRender.com. (B) Gel electrophoresis of multiplex PCR assay indicating flies that have lost one or both *Wolbachia* infections (*). The “synthetic positive” control was generated by combining previously generated *w*Ha and *w*No amplicons in equimolar ratios. Negative controls include flies cleared of their *Wolbachia* infections and no-template controls (NTC). (C) Pie chart summarizing the numbers of flies that lost *Wolbachia* infections (*n* = total 122 flies screened, *w*Ha only = 8, *w*No only = 1, uninfected = 5).

We hypothesized that we could perturb the transmission of the coinfection through a heat-mediated reduction in *Wolbachia* titers, which would facilitate a strong bottleneck and the opportunity to isolate individual *Wolbachia* strains. Indeed, subjecting coinfected flies to 30°C for 4 days resulted in some F1 progeny (11.5%) that were lacking in one or both *Wolbachia* strains (Fig. 5). This is in contrast to the offspring of coinfected flies reared at 25°C, where both *Wolbachia* strains are stably transmitted. In our routine lab screens, we have yet to find flies from this stock that do not carry both *Wolbachia* strains (*n* > 200 individuals).

## DISCUSSION

We hypothesized that the stability of multiple *Wolbachia* infections was made possible by some level of niche partitioning. The fact that many coinfections are comprised of more divergent strains with clade- or strain-specific genes (45–47) supports this idea. In *w*Ha and *w*No we identified strain-specific proteins predicted to be involved in separate metabolic pathways, as well as proteins that may provide different mechanisms for host interaction and virulence. Indeed, *w*Ha and *w*No have different patterns of tissue tropism across males and females, differentially respond to mating in a sex-dependent manner, and show different transmission and growth dynamics across fly development. The effect of mating on relative *Wolbachia* abundance might be driven by a range of factors. Physiological changes after mating could directly impact *Wolbachia* replication and titer but could also affect the number of host genome copies (e.g., nurse cell polyploidization, oocytes laid, or sperm transferred), which would affect the inference of relative titers. Absolute quantification and microscopy will be useful for teasing apart the interactions between physiology and *Wolbachia* infection density.

Strikingly, the relative abundance of *w*Ha and *w*No differed significantly between the ovaries and early embryos. However, the mechanisms that facilitate these patterns are still unclear. While *w*Ha and *w*No titers within the ovary are distinct from titers elsewhere in the body, there may be cell type specificity within the ovary. Ovaries contain a variety of both somatic and germ line cell types, and there are documented examples of cell type tropisms that also differ across *Wolbachia* strains (48, 49). Strain-specific imaging of whole ovarioles will allow us to determine how each *Wolbachia* strain is distributed within the ovary and in oocytes. The “assembly line” structure of *Drosophila* ovarioles offers a convenient way to capture changes in tissue specificity and titer that occur as eggs mature and may provide an explanation for the discrepancies in composition of the *Wolbachia* community that we see between whole ovaries and embryos. Additionally, the spatial distribution of *Wolbachia* within *Drosophila* embryos seems to follow a phylogenetic pattern. Supergroup B-type *Wolbachia* strains have a preference for cells in the anterior region of the embryo, while supergroup A-type strains have a heavier distribution in embryonic germ plasm (50). Differences in tissue tropism established early in embryogenesis may account for later differences across development and tissue. Strain-specific fluorescent imaging of whole ovarioles and embryos will offer insight into whether the distribution patterns identified by Veneti et al. (50) are maintained when there are multiple *Wolbachia* strains present.

After egg-lay and embryogenesis, the coinfection seems to converge on a pattern consisting of a relatively low and stable population of *w*No and a comparatively high level of *w*Ha that persists throughout development. When the adults emerge, we see the first evidence of increasing *w*No titers in females. Our data suggest that the switch from the high *w*Ha/*w*No ratio seen in juveniles to the relatively equal *w*Ha/*w*No titers of 3-day-old females occurs during adulthood, not metamorphosis. This process may be linked to ovary maturation as an adult rather than imaginal disc differentiation during the pupal period, but more in-depth analyses of the imaginal discs and the adult female maturation period are needed to tease this apart.

The differences in infection between ovaries and testes raise several questions about the reproductive manipulation induced by these strains: cytoplasmic incompatibility (CI). In the testes, CI results in altered sperm that cause embryonic lethality, unless “rescued” by a complementary infection in the oocyte (51). In the case of coinfections, typically each strain-specific alteration of the sperm requires a matching rescue or antidote in the embryo (10, 52), and previous studies indicate that *w*Ha and *w*No are not fully capable of rescuing the other strain’s CI induction (53). These CI induction and rescue processes are mediated by *Wolbachia* “Cif” proteins (CifA and CifB), and there is strong evidence that the level of Cif expression and the availability of strain-specific cognate partners are critical for proper induction and rescue (51, 54–57). Indeed, while *w*Ha and *w*No each encode a CifA and a CifB protein, their respective orthologs belong to different phylogenetic and functional subtypes (51, 56). Critically, the CifB proteins responsible for sperm modification have different enzymatic activities: *w*No’s B protein is a Cin type (CinB*^w^*^No^, a nuclease [58]), while *w*Ha’s B protein is a Cid type (CidB*^w^*^Ha^, a deubiquitylase [59]). Furthermore, both *in vitro* and *in vivo* approaches in these studies indicate that cognate CifA-CifB pairing is important for proper rescue in the embryo.

Given the importance of CifA-CifB cognate pairing and stoichiometry, it was interesting to find that the ratio of *w*Ha to *w*No within the testes was more variable between individuals than it was across ovaries (in which *w*Ha and *w*No titers were strongly correlated). Other studies in Drosophila melanogaster have shown that paternal grandmother age, which was not controlled for in our study, has a significant effect on CI strength in Drosophila melanogaster (60), which could correlate with *Wolbachia* titer and offer an explanation for the highly variable relative abundance of *Wolbachia* we observed in the testes. Additionally, *w*Ha was the dominant strain in testes, compared to *w*No, which was dominant in the ovaries. It is not clear if the ratios of *w*Ha and *w*No infections in the gonad tissues are reflective of the level of Cif proteins in gametes and ultimately the level of induction and rescue contributed by each strain.

It has been observed, however, that *Wolbachia* strains can differ in their tropism across males and females (61). Indeed, it is not clear if or how Cif expression would be regulated differently in instances where *Wolbachia* tropism differs between male and female reproductive tissues. Perhaps expression and deposition of Cif proteins are regulated in a cell-type-specific or coinfection-sensitive manner. Finally, we do not know if CI rescue is oocyte autonomous, or if Cif proteins are transported between cell types (e.g., from somatic follicle cells to the oocyte). Which cell types does *Wolbachia* need to be in, and at what time points in gametogenesis to cause or rescue CI? Perhaps the quantities of Cif proteins from each strain that are deposited in spermatozoa and oocytes are tightly regulated such that they more closely mirror each other. A combination of molecular approaches to assess Cif protein abundance in gametes and genetic tools to test for cell autonomy will be useful for understanding these processes and ultimately how CI is regulated.

Finally, we demonstrated that heat stress disrupts vertical transmission of *w*Ha and *w*No through an unknown mechanism. We hypothesize that heat stress negatively impacts *Wolbachia* titers (62), causing the bacteria to be “diluted” as cells in the ovary chain divide. In some instances, a developing oocyte will receive *Wolbachia* of only one strain or no *Wolbachia* at all. Using a heat treatment, we recovered more flies that had only the *w*Ha strain (and had lost *w*No) and only one example of a fly that had only *w*No (*n* = 1). This may be due to the preferential transmission of *w*Ha that we saw when comparing ovary and embryo coinfections or potentially strain-specific differences in heat sensitivity. Indeed, a recent study showed that temperature is a strong driver of *Wolbachia* transmission and spread at large scales (63), and there are many other examples of high temperatures that result in full or partial cures of *Wolbachia* (62). Our ability to segregate the strains into monoinfections in the same genomic background will be a useful tool for exploring the strain-specific contributions to host physiology and for understanding the interactions between coinfecting *Wolbachia* strains. Indeed, a combination of factors likely governs *Wolbachia* community dynamics, and it is unclear if *w*Ha and *w*No interactions with each other are competitive, synergistic, or perhaps parasitic. Disentangling the relative contributions of each strain to the stability of the coinfection will inform efforts to establish multiple infections of selected symbionts and contribute to understanding the dynamics of the intracellular community more broadly.

## MATERIALS AND METHODS

### Bioinformatics.

Protein sequences from the reference genomes of *w*Ha (GCF_000376605.1) and *w*No (GCF_000376585.1) annotated with PGAP (8, 64) were used to build orthologous groups of *Wolbachia* proteins using ProteinOrtho v5.15 with default parameters (65). Functional annotations were designated with BlastKOALA with (taxonomy group = bacteria) and (database = eukaryotes + prokaryotes) (66). A *Wolbachia* strain phylogeny was reconstructed with FtsZ sequences from A- and B-supergroup *Wolbachia* strains and a D-supergroup *Wolbachia* strain (*w*Bm) as outgroup (see Table S1 in the supplemental material). Amino acid sequences were aligned with MAFFT, and a simple neighbor joining (NJ) algorithm was used to reconstruct relationships including a JTT substitution model and 100 bootstrap replicates (67). Tree topology was visualized in FigTree v.1.4.4 (https://github.com/rambaut/figtree) prior to annotation in Inkscape v.1.1.2 (https://inkscape.org/) (67).

### Fly husbandry.

Fly stocks were maintained on standard Bloomington Drosophila Stock Center (BDSC) cornmeal agar medium (Nutri-Fly Bloomington formulation) at 25°C on a 24-h, 12:12 light-dark cycle under density-controlled conditions and 50% relative humidity. Experiments used the Drosophila simulans genome reference line (Cornell Stock Center SKU: 14021-0251.198), originally from Noumea, New Caledonia, which is stably coinfected with the *w*No and *w*Ha *Wolbachia* strains (15). We generated a *Wolbachia*-free stock with antibiotics for use as a negative control. This stock was generated by tetracycline treatment (20 μg/mL in the fly food for three generations), followed by reinoculation of the gut microbiome by transfer to bottles that previously harbored male flies from the original stock that had fed and defecated on the medium for 1 week (68). Gonad dissections were performed on live anesthetized flies under sterile conditions, and tissues were immediately flash frozen and stored at −80°C for later processing. Embryo collections and developmental synchronization were performed using timed 2-h egg-lays in mating cages on grape agar plates streaked with yeast paste. For developmental time points, single embryos were collected at 2 and 10 h, and the remaining embryos were transferred to BDSC medium, after which single flies were collected as L1, L2, and L3 larvae, white prepupae, red-eye bald pupae, and pharate males and females (less than 2 h postemergence). For comparing mated with unmated flies or tissues, all individuals were collected at the same time as pharate adults from density-controlled conditions and either mated or not mated 24 h postcollection. Three-days postcollection (2 days postmating) flies were dissected and/or flash frozen and stored at −80°C for later processing as described above.

### *Wolbachia* screening.

Infection status of all stocks was regularly screened with a multiplex PCR assay that produces size-specific amplicons for *w*Ha and *w*No (53). This PCR assay was also used in determining strain segregation during the differential curing experiments (see below). In all cases, DNA was extracted from individual flies with the Monarch genomic DNA purification kit (New England Biolabs), PCR assays were performed with the strain-specific multiplex primers from reference 53 and Q5 Hot Start High-Fidelity 2× master mix (New England Biolabs) in 20-μL reaction mixtures, and products were run on a 1% agarose gel, stained postelectrophoresis with GelRed (Biotium). For samples that screened negative for *Wolbachia*, DNA integrity was confirmed with PCR using general primers that target arthropod 28S (6). All primer sequences are listed in Table 1.

**TABLE 1:** Primer sequences used in this study

Assay target	Primer	Sequence (5′ to 3′)	Reference
*wsp* multiplex	81F	TGGTCCAATAAGTGATGAAGAAAC	James et al., 2002 (53)
	463R	TACCATTTTGACTACTCACAGCG	
	635R	GATCTCTTTAGTAGCTGATAC	

Arthropod 28S	28S_F	CCCTGTTGAGCTTGACTCTAGTCTGGC	Werren et al., 1995 (6)
	28S_R	AAGAGCCGACATCGAAGGATC	

*w*Ha *wsp* qPCR	wsp_wHa_qPCR_F	AAAGAAGACTGCGGATACTGAT	This study
	wsp_wHa_qPCR_R	CTGCGAATAAAGCCCTTCAAC	

*w*No *wsp* qPCR	wsp_wNo_qPCR_F	CAGCAATCCTTCAGAAGCTAGT	This study
	wsp_wNo_qPCR_R	AAATAACGAGCACCAGCATAAAG	

*D. simulans rpl32* qPCR	rpl32_Dsim_qPCR_F	AGGGTATCGACAACAGAGTG	This study
	rpl32_Dsim_qPCR_R	GGAACTTCTTGAATCCGGTG	

### Strain-specific qPCR.

To quantify the relative abundance of individual *Wolbachia* strains, we designed *w*Ha- and *w*No-specific quantitative PCR (qPCR) primer sets targeting unique ~100-bp amplicons of the *Wolbachia* surface protein (*wsp*). Assay specificity was verified with Sanger sequencing of amplicons, combined with validation against monoinfected samples generated during differential curing (see below). DNA was extracted from flies/tissues with the Monarch genomic DNA purification kit (New England Biolabs). Strain-specific abundance was assessed with the Luna universal qPCR master mix (New England Biolabs) following the manufacturer’s instructions and normalization to host genome abundance via amplification of *rpl32*. All reactions were run in technical triplicate alongside a standard curve and negative controls on a QuantStudio 3 real-time PCR system (Applied Biosystems). All primer sequences are listed in Table 1.

### Differential curing of *Wolbachia* strains.

To disrupt coinfection transmission, we designed a partial heat cure to reduce *Wolbachia* titers and increase the severity of the bottleneck as *Wolbachia* bacteria are deposited in each embryo. Bottles of ~200 Drosophila simulans flies were kept at 30°C for 4 days (or at 25°C as a control), after which flies were transferred to fresh medium under standard rearing conditions (see above) and allowed to oviposit for 3 days. Offspring (adults <24 h posteclosion) of the heat-treated mothers were collected and stored in ethanol for further processing, including DNA extraction and testing *Wolbachia* coinfection status following protocols detailed above.

### Statistics and data visualization.

All statistics and data visualization were carried out in R version 3.5.0 (69). We used permutational multivariate analysis of variance with the adonis function from the vegan package (70) to assess variation in coinfection titers (a multivariate response) across fly samples using Euclidean distance and 1,000 permutations. Fixed effects were specific to each experimental analysis and included: sex, mating status, and the interaction of the two (Fig. 2A); tissue, sex, and the interaction of the two (Fig. 2B); or developmental stage (Fig. 3). *Post hoc* comparisons were performed with either a Mann-Whitney U test (for pairwise comparisons, function “wilcox.test”) or a Kruskal-Wallis test (for >2 groups, function “kruskal.test”) followed by Bonferroni corrections in the case of multiple testing. Corrected *P* values are reported throughout as “corr.*P*.” In the case of the mated versus unmated ovary samples (Fig. 4A), we were interested in strain-specific dynamics upon mating, so we assessed variation in strain titers with a two-way analysis of variance (ANOVA) (function “aov”) including “strain” and “mated status,” along with their interaction, as fixed effects. Correlation between relative abundances of strains or between relative abundances in different tissues was assessed with a Spearman rank correlation for the data in Fig. 2 (function “cor.test,” method = “spearman”). Linear regression was performed with the “lm” function.
