# Supplementary material for: Infection Dynamics of Cotransmitted Reproductive Symbionts Are Mediated by Sex, Tissue, and Development
Source: Appl Environ Microbiol. 2022 Jun 22;88(13):e00529-22. doi: 10.1128/aem.00529-22 (PMC9275221; doi:10.1128/aem.00529-22)
Supplement: Supplemental file 2 — Fig. S1 and S2. Download aem.00529-22-s0002.pdf, PDF file, 0.5 MB [file aem.00529-22-s0002.pdf]

Supplemental figures for:

Infection dynamics of co-transmitted reproductive symbionts are mediated by sex, tissue, and development

Megan W Jones[1], Laura C Fricke[1], Cody J Thorpe[1], Lauren O Vander Esch[1], Amelia RI Lindsey[1*]

[*]To whom correspondence should be addressed (alindsey@umn.edu)
[1]Department of Entomology, University of Minnesota, St. Paul, Minnesota, 55108

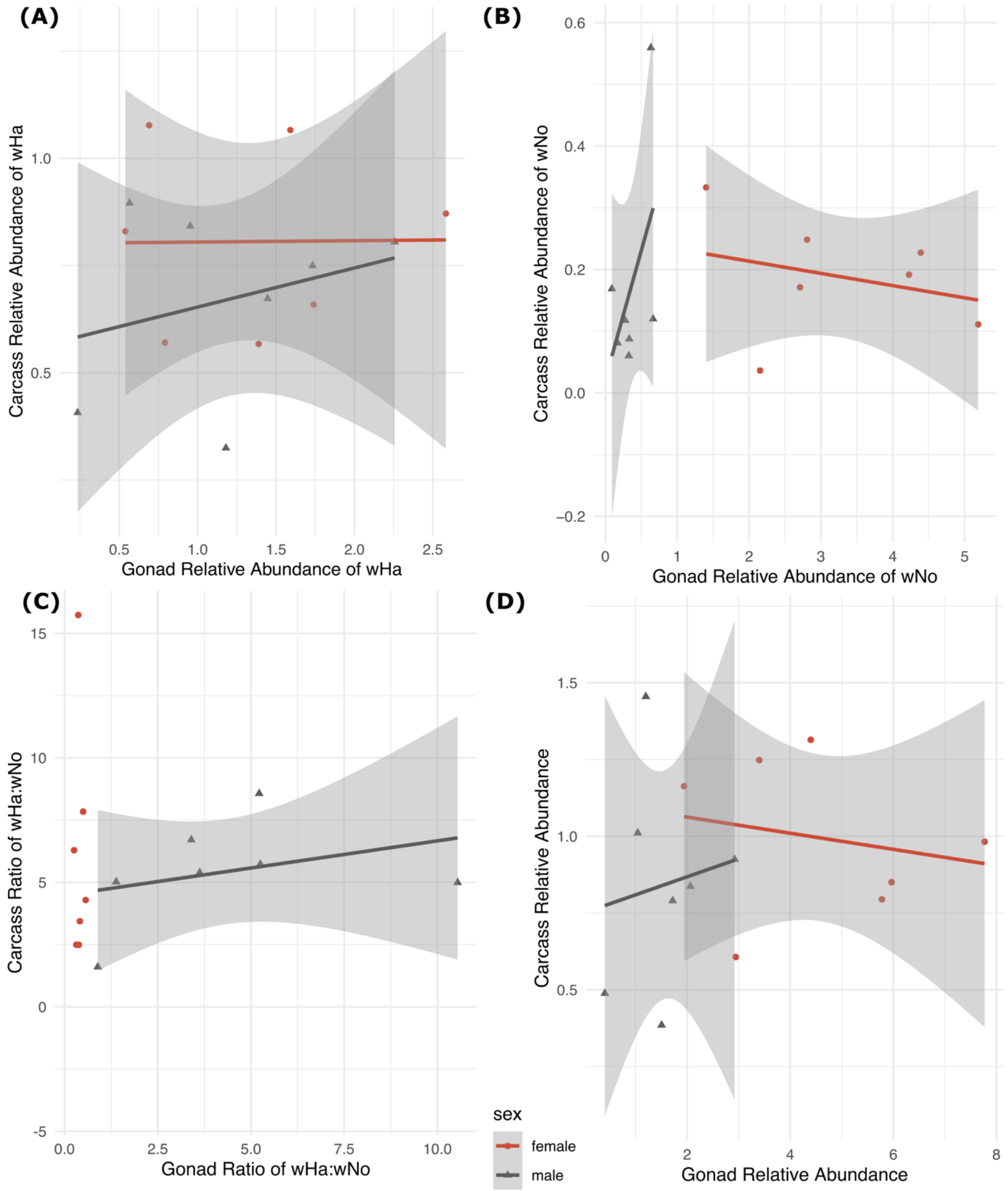

Supplemental Figure S1. **Within-fly gonad and carcass infection dynamics.** There is no correlation between the relative abundance of *w*Ha **(A)** or *w*No (**B)** across gonads and carcasses. (*w*Ha females: rho = -0.0357, p = 0.9635; *w*No females: rho = -0.2143, p = 0.6615; *w*Ha males: rho = 0, p = 1; *w*No males: rho = 0.2143, p = 0.6615). **(C)** There is no correlation between the

*w*Ha:*w*No ratio in gonads and carcasses (females: rho = 0.0357, p = 0.9635; males: rho = 0.2857, p = 0.556). Linear regression is only shown for males, as there is limited variation in the x-axis for females. **(D)** There is no correlation between the total abundance of *Wolbachia* in gonads and carcasses (females: rho = -0.1071, p = 0.8397; males: rho = 0.1736, p = 0.5526).

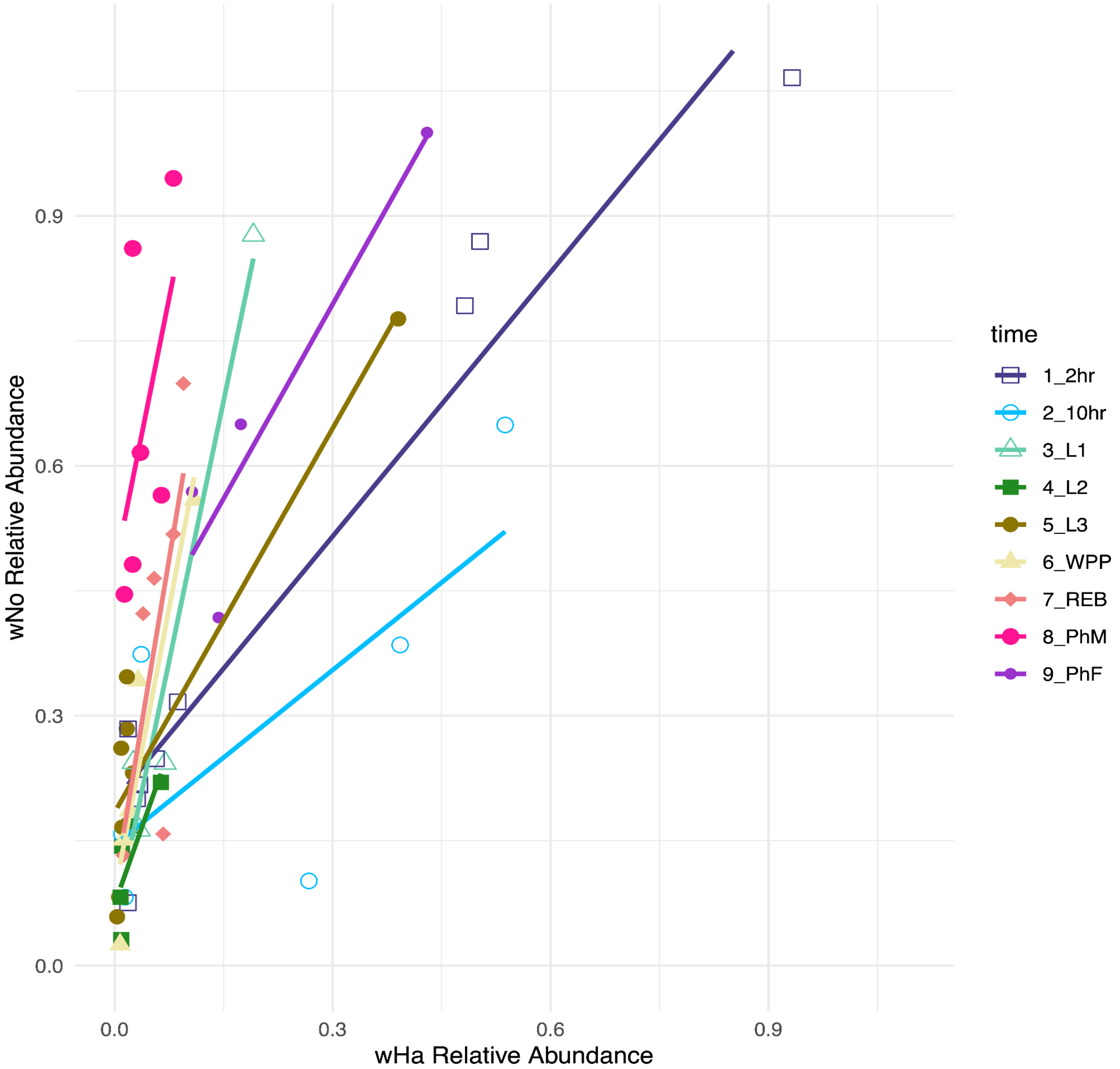

Supplemental Figure S2. ***w*Ha and *w*No correlation across development.** Relationship between *w*Ha and *w*No relative abundance across flydevelopment.
